# Experimental Characterization of *Cis*-Acting Elements Important for Translation and Transcription in Halophilic Archaea

**DOI:** 10.1371/journal.pgen.0030229

**Published:** 2007-12-21

**Authors:** Mariam Brenneis, Oliver Hering, Christian Lange, Jörg Soppa

**Affiliations:** Institute for Molecular Biosciences, Goethe-University, Frankfurt, Germany; Stanford University, United States of America

## Abstract

The basal transcription apparatus of archaea is well characterized. However, much less is known about the mechanisms of transcription termination and translation initation. Recently, experimental determination of the 5′-ends of ten transcripts from Pyrobaculum aerophilum revealed that these are devoid of a 5′-UTR. Bioinformatic analysis indicated that many transcripts of other archaeal species might also be leaderless. The 5′-ends and 3′-ends of 40 transcripts of two haloarchaeal species, Halobacterium salinarum and *Haloferax volcanii,* have been determined. They were used to characterize the lengths of 5′-UTRs and 3′-UTRs and to deduce consensus sequence-elements for transcription and translation. The experimental approach was complemented with a bioinformatics analysis of the H. salinarum genome sequence. Furthermore, the influence of selected 5′-UTRs and 3′-UTRs on transcript stability and translational efficiency in vivo was characterized using a newly established reporter gene system, gene fusions, and real-time PCR. Consensus sequences for basal promoter elements could be refined and a novel element was discovered. A consensus motif probably important for transcriptional termination was established. All 40 haloarchaeal transcripts analyzed had a 3′-UTR (average size 57 nt), and their 3′-ends were not posttranscriptionally modified. Experimental data and genome analyses revealed that the majority of haloarchaeal transcripts are leaderless, indicating that this is the predominant mode for translation initiation in haloarchaea. Surprisingly, the 5′-UTRs of most leadered transcripts did not contain a Shine-Dalgarno (SD) sequence. A genome analysis indicated that less than 10% of all genes are preceded by a SD sequence and even most proximal genes in operons lack a SD sequence. Seven different leadered transcripts devoid of a SD sequence were efficiently translated in vivo, including artificial 5′-UTRs of random sequences. Thus, an interaction of the 5′-UTRs of these leadered transcripts with the 16S rRNA could be excluded. Taken together, either a scanning mechanism similar to the mechanism of translation initiation operating in eukaryotes or a novel mechanism must operate on most leadered haloarchaeal transcripts.

## Author Summary

Expression of the information encoded in the genome of an organism into its phenotype involves transcription of the DNA into messenger RNAs and translation of mRNAs into proteins. The textbook view is that an mRNA consists of an untranslated region (5′-UTR), an open reading frame encoding the protein, and another untranslated region (3′-UTR). We have determined the 5′-ends and the 3′-ends of 40 mRNAs of two haloarchaeal species and used this dataset to gain information about nucleotide elements important for transcription and translation. Two thirds of the mRNAs were devoid of a 5′-UTR, and therefore the major pathway for translation initiation in haloarchaea involves so-called leaderless transcripts. Very unexpectedly, most leadered mRNAs were found to be devoid of a sequence motif believed to be essential for translation initiation in bacteria and archaea (Shine-Dalgarno sequence). A bioinformatic genome analysis revealed that less than 10% of the genes contain a Shine-Dalgarno sequence. mRNAs lacking this motif were efficiently translated in vivo, including mRNAs with artificial 5′-UTRs of total random sequence. Thus, translation initiation on these mRNAs either involves a scanning mechanism similar to the mechanism operating in eukaryotes or a totally novel mechanism operating at least in haloarchaea.

## Introduction

Determination of the 5′- and 3′-ends of transcripts is used to identify the points of transcription initiation and termination in order to gain important information about both processes. In fact, determination of transcript 5′-ends led to the conclusion that archaea have basal promoter elements that differ from bacterial promoters and resemble eukaryotic polymerase II promoters, thus underscoring the view that archaea are a third domain of life [1]. A survey of all experimentally determined 5′-ends led to the conclusion that all archaeal genes share the existence of a TATA box and a newly discovered transcription factor B recognition element (BRE), but that the respective consensus sequences are not identical in different groups of archaea [2].

While the mechanism of transcription initiation is well-characterized [3–6], transcription termination in Archaea is far from being understood. Early results indicated that extended oligo-U or oligo-pyrimidine stretches are involved in termination [7–9]. Recently, a thorough study using an in vitro termination system has been performed that corroborated the importance of oligo-U stretches. However, it has also been shown that transcription termination in archaea is more complicated than anticipated and a coherent picture could not be generated [10].

Experimental determination of 5′- and 3′-ends also enables the calculation of the lengths of 5′- and 3′-UTRs of transcripts. For a long time it was thought that, with very few exceptions, mRNAs of protein-coding genes in all organisms consist of a 5′-UTR, a coding region, and a 3′-UTR. UTRs can have various functions. They have been shown to influence transcript stability, polyadenylation, differential translational control, and intracellular localization [11–16]. Mutations in 3′-UTRs have been associated with human diseases [17]. Sequences regulating translation initiation are located in the 5′-UTRs, and two different mechanisms for determining the codon at which translation initiates have been identified in prokaryotes and eukaryotes. The major mechanism of prokaryotes relies on the interaction of a Shine-Dalgarno (SD) sequence (located a few nucleotides upstream of the coding region) with the 3′-end of the 16S rRNA, while eukaryotes use a scanning mechanism starting from the RNA 5′-end. The first sequences of archaeal transcript 5′-ends revealed that they did not have a 5′-UTR and thus were leaderless [18–20]. However, because “SD sequences” downstream of the start codon were proposed and subsequently characterized archaeal transcripts were found to have 5′-UTRs; this did not lead to a change of concept. The first proposition that two different mechanisms of translation initiation might operate in archaea was based on a bioinformatic analysis of 144 Sulfolobus solfataricus genes which indicated that distal genes in operons were preceded by a SD sequence but single genes and proximal operon genes were leaderless and thus have to use an alternative mechanism [21]. Supporting this view, experimental determination of 5′-ends of ten Pyrobaculum aerophilum transcripts showed that all of them were leaderless, and a concomitant genome analysis led to the suggestion that most, if not all transcripts, of single genes and proximal genes in operons are leaderless [22,23]. Bioinformatic analysis of 18 archaeal genomes led to the proposal that P. aerophilum is not exceptional and to the prediction that nine other species also contain a high fraction of leaderless transcripts (31%–74%) [24]. In two cases, experimental evidence for the efficient translation of leaderless transcripts in archaea were presented. Using an in vitro translation system of S. solfataricus it was shown that mutation of the native SD sequences of two genes resulted in a total lack of translation, but that deletion of the native 5′-UTR restored translation to a large extent [25]. Using an in vivo system it was shown for one Halobacterium salinarum gene that mutation of the native SD sequence decreased translation efficiency considerably, but that 5′-UTR deletion increased the protein level by more than 10-fold [26]. Reinforcing the idea that two different pathways are used, it was shown that the interaction between the transcript and the 30S ribosomal subunit differs for leadered SD-containing and leaderless transcripts [27].

The relative portions of leaderless and leadered transcripts in different archaea have been predicted in silico, but until now very few 5′-ends have been determined experimentally that could verify these predictions. As even fewer 3′-ends have been determined experimentally, the fraction of transcripts containing a 3′-UTR and the 3′-UTR length distribution are unknown. This cannot be predicted in silico because the archaeal terminator structure is not known. In addition, transcript 3′-ends might be modified posttranscriptionally, e.g., by polyadenylation,

In this work, we have determined the 5′- and 3′-ends of 40 transcripts from two species of halophilic archaea, H. salinarum and Haloferax volcanii. The two species belong to different genera and are dissimilar concerning cell morphology, optimal salt concentration, movement and chemotaxis, and energy metabolism. This collection of archaeal 5′- and 3′-ends was used to determine 5′- and 3′-UTR lengths and to gain information on the mechanisms of transcription initiation and termination and translation initiation. A reporter gene system and tag sequence fusions were used to experimentally characterize the in vivo function of selected 5′-UTRs and 3′-UTRs. In addition, the translational efficiencies of artificial 5′-UTRs were characterized. The experimental results were complemented by bioinformatic analyses of the H. salinarum genome.

## Results

### Determination of 5′- and 3′-Ends of Haloarchaeal Transcripts

**Figure 1:**
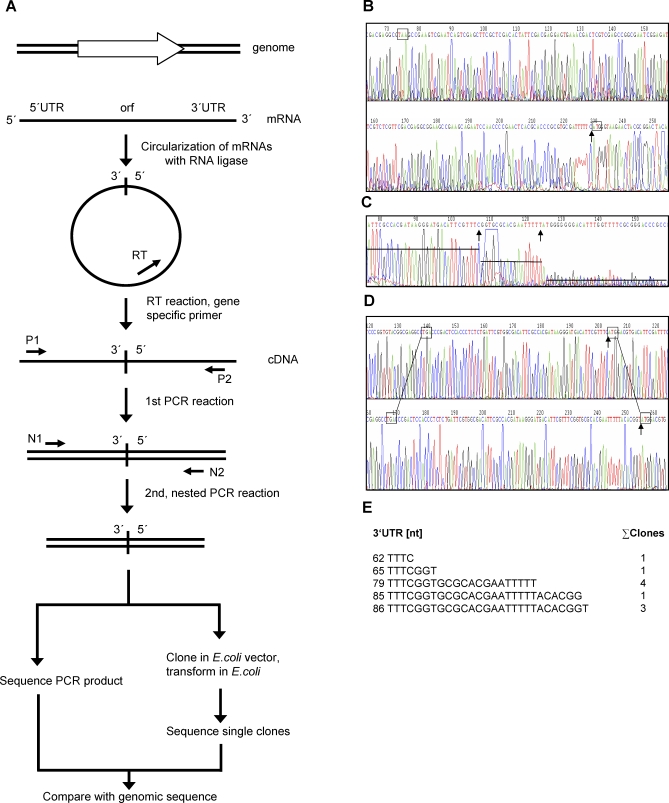
Determination of 5′- and 3′-Ends of Haloarchaeal Transcripts (A) Overview of the method. A recently developed method [28] was used to determine 5′-ends and 3′-ends of haloarchaeal transcripts. The overview schematically shows the different steps of the protocol. Circularization of transcripts with T4 RNA ligase is only possible if their 5′-ends are monophosphorylated, in contrast to the 5′- triphosphate that is present when they are newly synthesized. This led to the initial belief that only processed transcripts can be analyzed [28], but it turned out that the method is also ideally suited to characterize primary transcripts (compare Discussion). (B) Sequence of a PCR product representing a transcript with only one specific 3′-end (number 12 in Table 2). The stop and start codons of the gene are boxed, and the ligation point of the 5′-end and the 3′-end are denoted by an arrow. (C) Sequence of a PCR product representing a transcript with several 3′-ends (number 7 in Table 2). Different signal intensities are indicated by lines and the ligation point of the 5′-end and two different 3′-ends are denoted by arrows. (D) Sequences of two clones after cloning the PCR product shown in C (number 7 in Table 2). The results of two sequencing reactions of independent clones are shown. In both cases, the stop codon and start codon of the gene are boxed, and the ligation point of the 5′-end and the 3′-end are denoted by an arrow. (E) Results after sequencing ten clones. The sequences of five different 3′-ends and their number of occurrence are shown. The 5′-end was found to be identical in all ten cases.

A recently developed method [28] (Figure 1A) was used to determine 5′- and 3′-ends of 40 transcripts from the two haloarchaeal species H. salinarum and H. volcanii. In short, cellular RNA was isolated and circularized using T4 RNA ligase. The RNA of every gene of interest was transformed into cDNA using a gene-specific primer and reverse transcriptase. The DNA was amplified in two consecutive nested PCR reactions with four gene-specific primers, yielding a PCR product comprising the known 5′- and 3′-regions of the open reading frame and the unknown 5′- and 3′-UTRs of the transcript. Sequencing of the PCR product and comparison with the genomic sequence allowed the determination of 5′-end and 3′-end. An example is shown in Figure 1B. In several cases the signal strength of the sequence varied over the whole length of the PCR product, and one or several steps of decreasing signal intensity were observed, indicating that the 5′-end, the 3′-end, or both were not uniform (an example is shown in Figure 1C). In these cases the PCR product was cloned into an Escherichia coli vector and the sequences of ten clones were determined. It turned out that in all cases the 5′-ends of the clones were identical, whereas the 3′-ends varied. One representative example is illustrated in Figure 1D and 1E. The results revealed that transcription is initiated faithfully at only one specific nucleotide. The 3′-end variability of some transcripts might either be caused by termination at several points within a region of about 30 nucleotides or by differential degradation from the 3′-end.

**Table 2:**
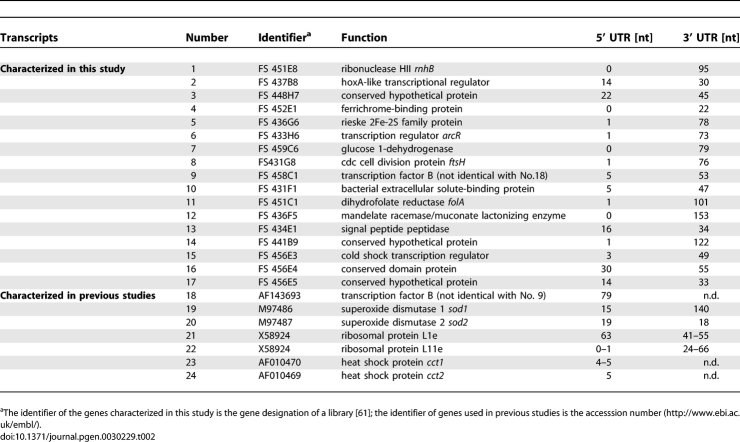
5′ UTRs and 3′ UTRs of Transcripts of *H. volcanii*

The method was used to analyze 40 transcripts from H. salinarum or H. volcanii, thus generating by far the largest database of 5′- and 3′-ends of archaeal transcripts available. The genes were selected in order to exemplify many different functional categories (e.g., energy metabolism, anabolic metabolism, gene regulation, translation, stress response, proteins of unknown function), expression levels (e.g., ribosomal protein, transcriptional regulator), and cellular localizations (cytoplasmic, membrane-bound, extracellular). The results for the 40 genes are summarized in Tables 1 and 2. The EMBL nucleotide sequence database (http://www.ebi.ac.uk/embl/) and the NCBI literature database (http://www.pubmed.de/data/nlm.link.html) were searched for previously determined 5′- and 3′-ends of transcripts from both species and all publicly available examples were also included (Tables 1 and 2).

**Table 1:**
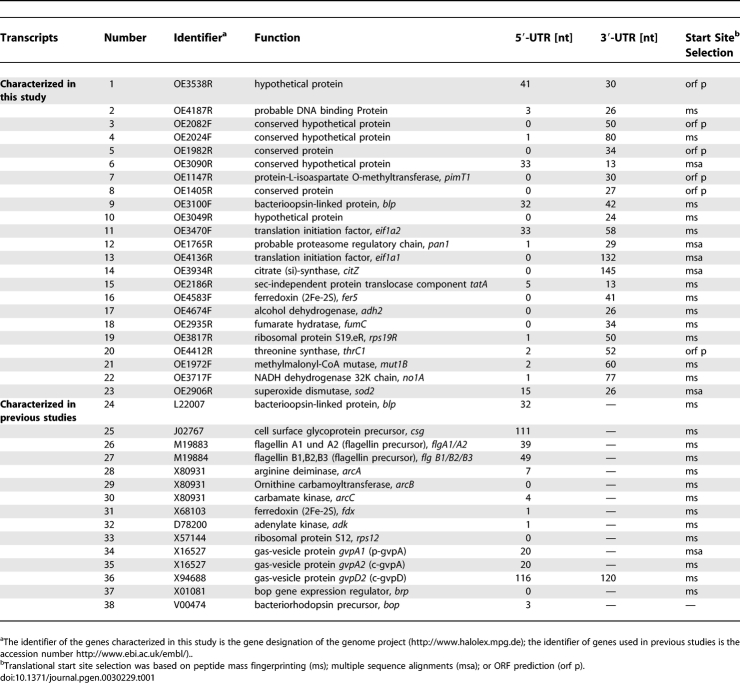
5′-UTRs and 3′-UTRs of Transcripts of H. salinarum

### Promoter Structure in Haloarchaea

**Figure 2:**
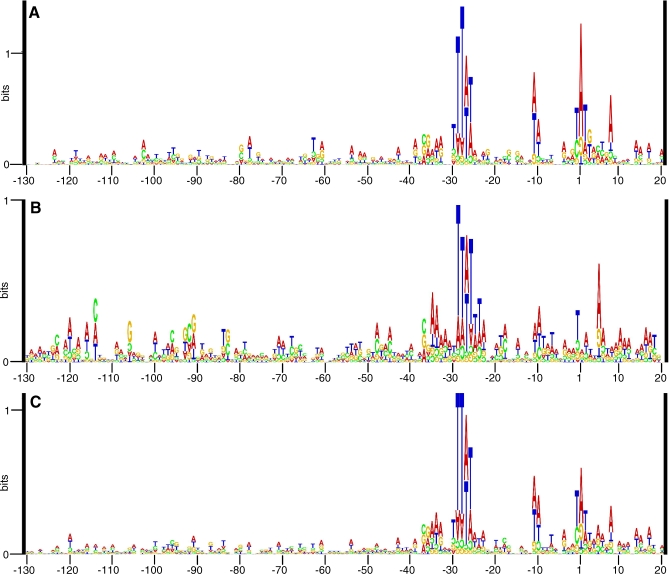
Basal Promoter Elements Identified Upstream of 63 Haloarchaeal Genes The sequences from −130 to +20 around the experimentally determined 5′-ends of haloarchaeal transcripts (Tables 1 and 2) were retrieved from databases. Sequence logos were constructed with the program “RNA structure logo” [29]. For all logos, the height of the upright letters corresponds to the degree of conservation of that nucleotide at the respective site, measured in bits. The upside down letters indicate that the occurrence of the respective nucleotide is lower than expected in random sequences of the same GC content, and the height corresponds to the degree of underrepresentation. (A) Comparison of all 37 genes of H. salinarum (Table 1). (B) Comparison of all 26 genes of H. volcanii (Table 2). (C) Comparison of all 63 haloarchaeal genes (Tables 1 and 2).

In order to characterize haloarchaeal basal promoter elements, the sequences from −130 nt to +20 nt (relative to the experimentally determined 5′-ends) were extracted from the genome database of H. salinarum (http://www.halolex.mpg.de) and partial genome database of H. volcanii (http://www.tigr.org). Sequence logos [29,30] were generated to identify potentially functional DNA motifs present at a fixed distance from the transcription initiation point. Three sequence logos were generated, two species-specific logos for all genes of H. salinarum and H. volcanii with known 5′-ends (Figure 2A and 2B, respectively), and one that combined the data for both species (Figure 2C). Clearly, the most prominent promoter element is the TATA box, centered at position −27/−28, with the consensus sequence “TTWT.”

The “transcription factor B recognition element” (BRE) is conserved upstream of the TATA box (Figure 2). Its consensus sequence “CGAAA” is extended compared to the “AA” motif discovered in two previous studies [2,22] and to the bioinformatic analysis of the H. salinarum genome (see below).

The promoter analysis led to the detection of a “WW” element at positions −10 and −11 (Figure 2). The conserved spacing to the TATA box, the BRE, and the transcription start site strongly indicates that this novel element is a bona fide basal promoter element. It remains to be discovered which protein makes contact to these two bases. Likely candidates are TFB and the RNA polymerase.

It is known that the basal promoter elements have an optimal spacing to the transcriptional start site, e.g., the TATA box is centered at −27/−28, although a small degree of variation seems to be possible. Shifting of the TATA box by one or two nucleotides was reported to enhance the consensus sequence in a previous study [2]. Therefore, the upstream sequences of the genes summarized in Table 1 were inspected individually, and it was investigated whether sequence shifting would improve the alignment. However, this was not the case (unpublished data).

### Transcription Termination in Haloarchaea

**Figure 3:**
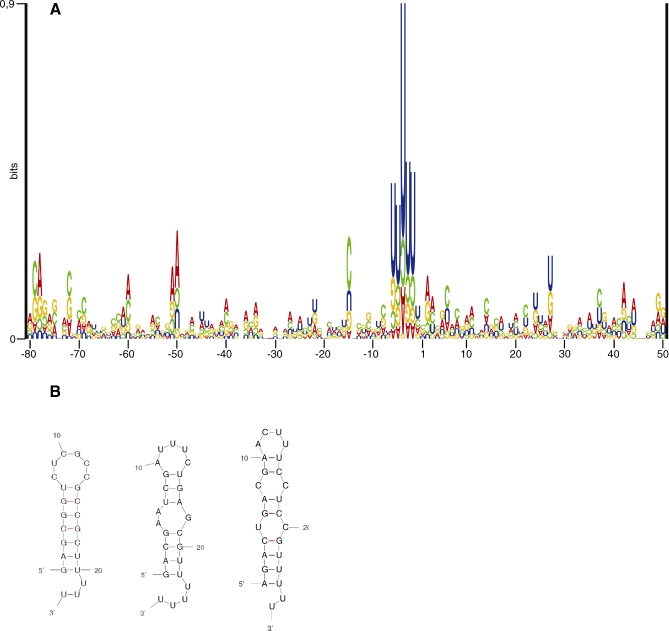
Transcription Termination in Halophilic Archaea (A) The sequences from −80 to +50 around the experimentally determined 3′-ends of haloarchaeal transcripts (Tables 1 and 2) were retrieved from databases. A sequence logo was constructed with the program “RNA structure logo” [29]. (B) Three typical examples of secondary structures predicted to be formed at the 3′-end of transcripts. Note that the oligo-U motif is partially involved in basepairing in the stemloop in all three cases. From left to right, the examples show the 3′-ends of the transcripts of the genes numbers 4, 15, and 16 in Table 2.

To identify sequence motifs important for termination of transcription, the last 80 nt of the transcribed DNA and the 50 nt downstream of the termination site were retrieved for all genes with experimentally determined 3′-ends and a sequence logo was generated (Figure 3A). A motif of five Us close to the 3′-end is strongly conserved. Apart from that, no conserved sequence motif was found either upstream or downstream of the termination site. To identify structural conservations, all 3′-UTRs were subjected to in silico folding using three different programs (see Materials and Methods). More than 80% of the transcripts were found to contain a putative stemloop structure at their 3′-ends, preceeding the penta-U motif or involving some of the Us (Table 1: transcripts 1–23 with the exception of 4 and 15; Table 2: transcripts 1–17 with the exception of 2, 5, 8, and 11). Three typical examples are shown in Figure 3B, indicating that the structure of the putative stemloops is not uniform but might include one or two bulges. Taken together, the observations suggest that transcriptional termination in haloarchaea occurs at one or several sites located downstream of a pentaU motif that is often preceded by a putative stemloop unconserved in sequence and structure.

Analysis of the 3′-ends of the 40 haloarchaeal transcripts revealed that they are identical to the genome sequence and in neither species extra nucleotides were added after transcription. This observation corroborates a recent study that did not find any evidence for polyadenylation using bulk RNA of H. volcanii [31] and extends the analysis to a second haloarchaeal species, H. salinarum.

### 3′-UTRs: Lengths, Putative Structural Elements, and Possible Roles

All 40 haloarchaeal transcripts were found to have a 3′-UTR (Tables 1 and 2). The GC content of the 3′-UTRs was the same as that of the coding regions and no differences between the two species were detected. The lengths vary from 13 to 154 nt with an average length of 57 nt (median 49 nt). 80% of the 3′-UTRs have a length between 20 and 80 nt, and only seven 3′-UTRs are longer than 100 nt.

The program MEME [32] was used to search for conserved sequence motifs in different subsets of 3′-UTRs (e.g., in the UTRs of all genes, of the H. salinarum, and of the H. volcanii genes). In each case, the oligo-U motif was involved in transcription termination (Figure 3A) but no additional sequence motif was retrieved. Three different programs (see Materials and Methods) were used to predict the secondary structure of the 3′-UTRs either on its own or in the context of the whole mRNA. However, no conserved structural motifs apart from the stemloop structures preceding the penta-U motif (see above) could be detected.

### Leaderless versus Leadered Transcripts and the Mechanism of Translation Initiation

Leaderless transcripts contain none or only a few nucleotides upstream of the translational start codon while the 5′-UTR of leadered transcripts needs to be long enough to contain a Shine-Dalgarno (SD) sequence. It has been shown that there must be at least four nucleotides between the 3′-end of the SD sequence and the start codon in H. volcanii [26], and that a SD sequence consists of at least five nucleotides (see below). Therefore we categorized all transcripts with a 5′-UTR of 10 nt or more as “leadered,” and all transcripts with fewer nucleotides as “leaderless.” In both species, about two thirds of all transcripts are leaderless, i.e., 26 of 37 H. salinarum transcripts (Table 1, *blp* was counted only once) and 15 of 24 H. volcanii transcripts (Table 2). On average, the 5′-UTRs of leadered transcripts are longer in H. salinarum than in H. volcanii (median 33 versus 19 nt). It remains to be discovered whether these differences are caused by the small sets of investigated genes of both species or if they reflect differences of the translation initiation process. The 5′-UTRs in both species are shorter than those in eukaryotes, which have an average length of about 100 nt [13].

**Figure 4:**
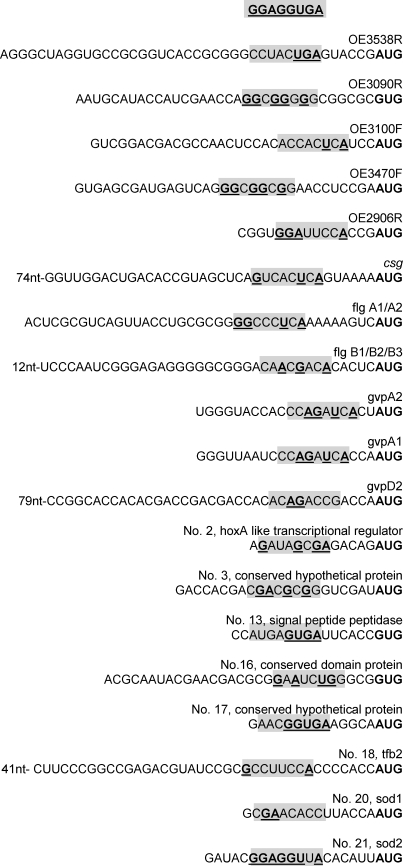
5′-UTRs of Leadered Transcripts of H. salinarum and H. volcani The sequences of the 5′-UTRs including the translation initiation codon of all leadered transcripts are shown (Tables 1 and 2). The sequence of eight nucleotides complementary to the 3′-end of the 16S rRNA is shown on top of the figure. In E. coli the optimal distance between the 3′-edge of the SD sequence and the A of the start codon is 5 nt and 85% of all genes have distances between 3 and 7 nt (both included) [54]. Because the optimal distance in haloarchaea is not known, the region that fits best to the complementary region was searched allowing distances between 2 and 10 nt. The best matching region is shaded, and the nucleotides that could base-pair with the 16S rRNA are shown in bold and are underlined. The translational start codons are shown in bold.

Translation initiation on leadered haloarchaeal transcripts was thought to depend on the “conventional” pathway, and thus the 5′-UTRs were expected to contain SD sequences. For reasons described below a SD sequence was defined to consist of at least five consecutive nucleotides able to hybridize to the 3′-end of the 16S rRNA and to have a distance between three and seven nucleotides between its 3′-edge and the start codon. Surprisingly, most haloarchaeal 5′-UTRs were devoid of a SD sequence, and only two out of 19 leadered transcripts harbored a predicted SD sequence (Figure 4). A more relaxed criterion of four consecutive nucleotides and a distance of zero to nine nucleotides led to the inclusion of just one further gene, but four nucleotides are very unlikely to function as a SD sequence in haloarchaea (see Discussion).

It is unclear how translation is initiated on SD-less leadered transcripts, because the “leaderless pathway” cannot be used by leadered transcripts, and leadered transcripts are thought to need the interaction of a SD sequence with the 3′-end of the 16S rRNA. Therefore, it seemed important to characterize experimentally whether or not SD-less leadered transcripts are translated efficiently in vivo.

### Experimental Characterization of In Vivo Functions of Selected 5′-UTRs and 3′-UTRs Using a Reporter Gene System and Gene Fusions

To address translational efficiencies experimentally, the steady state levels of transcripts and their encoded gene products need to be quantitated. However, no quantitative assays for most of the gene products summarized in Tables 1 and 2 were available. Two different approaches were used to characterize the in vivo function of UTRs in H. volcanii and H. salinarum.

**Figure 5:**
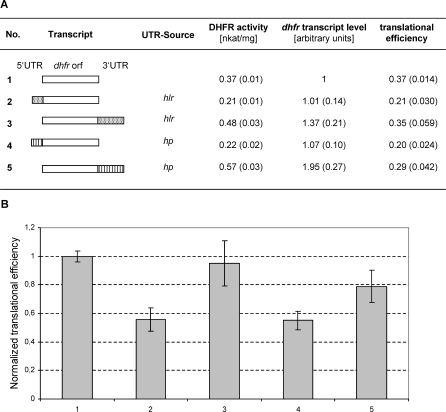
Characterization of the In Vivo Function of UTRs in H. volcanii Using a Reporter Gene (A) The *dhfr* reporter gene and the gene fusions containing 5′-UTRs and 3′-UTRs of genes number 2 and 3 of Table 2 are shown schematically. The genes encode a “HoxA-like transcriptional regulator” (*hlr*, number 2 in Table 2) and a “conserved hypothetical protein” (*hp*, number 3 in Table 2). The DHFR enzymatic activity, the *dhfr* transcript level, and the translational efficiency are tabulated. Three biological replicates were perfomed and average values were calculated. The numbers in parentheses are standard deviations. The translational efficiencies were calculated by dividing the DHFR activity by the *dhfr* transcript level. (B) The translational efficiency after normalization to the control transcript is visualized.

A reporter gene system was established for H. volcanii. The *H. volcanii dhfr* gene was chosen as a well-established assay that has been successfully used as a reporter gene for in vivo promoter activity [33]. The *dhfr* transcript is leaderless. A variant of the gene without its native 3′-UTR was generated and used as a control. Two genes encoding a “HoxA-like regulator” (*hlr*) and a “conserved hypothetical protein” (*hp*) were chosen for the investigation of the in vivo roles of UTRs (numbers 2 and 3 in Table 2). The native transcripts of both genes contain a 5′-UTR lacking a SD sequence. Four plasmids were generated that encode transcripts containing either the 5′-UTR or the 3′-UTR of the two genes fused to the *dhfr* open reading frame (Figure 5A). H. volcanii cultures containing the respective constructs were grown to the exponential growth phase (2 × 10^8^ cells/ml) and the DHFR activities as well as the *dhfr* transcript levels were determined quantitatively (Figure 5A).

The determination of the average transcript level showed that the fusion of 5′-UTRs to the control transcript did not alter the stability of the transcript. In contrast, fusion of the 3′-UTRs to the control transcript in both cases enhanced transcript abundance by a factor of 1.4 and 2, respectively (numbers 3 and 5 in Figure 5A). Different transcription initiation rates can be excluded as the 5′-parts of both transcripts are identical to the control transcript, therefore it can be inferred that the transcripts have different stabilities. Apart from the discovery that 3′-UTRs are involved in selenocysteine incorporation in some methanogenic archaea [34], this enhancement of transcript stability is the second experimentally proven biological function of 3′-UTRs in archaea, and this role is probably of much more general relevance.

The DHFR activities and transcript levels were used to calculate translational efficiencies of the five transcripts under investigation (Figure 5A). The results were normalized to the translational efficiency of the control transcript and are summarized in Figure 5B. In both cases, the fusion of a 5′-UTR to the leaderless transcript led to a decrease in translational efficiency by a factor of about two (Figure 5B, numbers 2 and 4). This underscores that leaderless transcripts do not only form the majority of transcripts but that they are very efficiently translated in haloarchaea, in contrast to Escherichia coli [35]. For the first time the results prove experimentally that transcripts with a SD-less 5′-UTR are translated in haloarchaea.

In one case fusion of 3′-UTRs to the *dhfr* transcript had no effect on translational efficiency (Figure 5B, number 3); in the second example it led to a slight decrease (Figure 5B, number 5).

**Figure 6:**
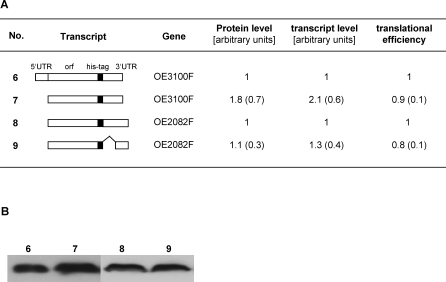
Characterization of the In Vivo Function of UTRs in H. salinarum Using Gene Fusions (A) The constructs that were integrated into the genome of H. salinarum are shown schematically. Protein and transcript levels were determined using a anti-his_6_-tag antibody and qRT-PCR, respectively. The translational efficiencies were calculated by dividing the protein levels by the transcript levels. Three biological replicates were performed and the average values and standard deviations (in parentheses) were calculated. (B) Representative western blot analysis of cultures carrying the constructs shown in (A).

In order to determine the in vivo function of UTRs in H. salinarum, a different strategy was chosen, i.e., the addition of a hexahistidine (his_6_) tag to the respective proteins; thus enabling us to quantify the levels of different proteins using the same commercial anti-his-tag antibody. Two genes were chosen for the analysis, one gene encoding a SD-less leadered transcript (OE3100F, number 9 in Table 1) and one gene encoding a leaderless transcript with a long 3′-UTR (OE2082F, number 3 in Table 1). His-tagged control versions as well as his-tagged versions carrying a deletion of the 5′-UTR or a deletion within the 3′-UTR were integrated into the chromosome of H. salinarum (Figure 6). Exponentially growing cultures (2 × 10^8^ cells/ml) were used to determine transcript levels by quantitative reverse transcriptase PCR (qRT-PCR) and protein levels with an anti-his_4_-tag antibody. Unexpectedly, the transcript level of the deletion variant was elevated about 2-fold suggesting that the 5′-UTR destabilizes the transcript or that the transcription rate is enhanced in the mutant. However, it turned out that the transcript carrying a SD-less leader sequence, as well as the transcript lacking its native 5′-UTR, was translated with very similar or identical efficiency (Figure 6). Deletion of more than half of the 3′-UTR of the OE2082F transcript had no effect on transcript stability or translation efficiency.

Taken together, the results demonstrate that leaderless transcripts, as well as leadered transcripts without a SD sequence, are efficiently translated in vivo in both haloarchaeal species.

### Translational Efficiencies of Transcripts with Artificial 5′-UTRs of Random Sequence

**Figure 7:**
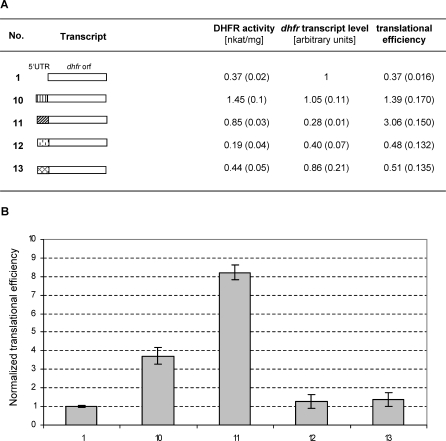
Translational Efficiencies of Artificial UTRs of Random Sequences in H. volcanii Four 5′-UTRs of random sequence were generated and cloned upstream of the reporter gene *dhfr.* (A) The transcripts are shown schematically. The DHFR activities, the transcript levels and the translational efficiencies are tabulated. Three biological replicates were performed and average values and standard deviations (in parentheses) were calculated. (B) The translational efficiencies are shown after normalization to the control transcript devoid of a 5′-UTR. (C) The sequences of the four random 5′-UTRs are tabulated including the start codon (bold) are shown. On top, the sequence of eight nucleotides that are complementary to the 3′-end of the 16S rRNA is shown. The optimal spacing between SD sequence and start codon in archaea is unknown, therefore the optimal spacing determined with E. coli has been chosen.

Bioinformatic analysis of SD-less 5′-UTRs did not identify consensus sequences or conserved structural elements possibly involved in translation initiation (unpublished data). Therefore we tested whether artificial 5′-UTRs of random sequence have the ability to initiate translation in vivo. Four random sequences of 20 nt (equivalent to the average length of natural 5′-UTRs in H. volcanii) were generated and fused to the *dhfr* reporter gene. Very unexpectedly, translational efficiencies of all four transcripts were at least as high as of the leaderless control transcript and the efficiencies of two transcripts were even 4-fold to 8-fold higher (Figure 7). The 8-fold difference in translational efficiencies indicates that the random sequences contain as yet unrecognized sequence or structural information that influences translation initiation. Nevertheless, the results of all four artificial 5′-UTRs of random sequence underscore that translation was efficiently initiated at leadered transcripts devoid of a SD sequence.

### Genome-Wide Bioinformatic Analysis of Translation Initiation

**Figure 8:**
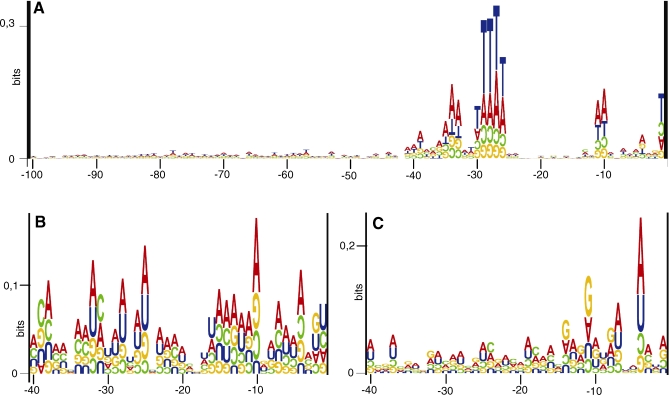
Bioinformatic Analysis of the H. salinarum Genome (A) Sequence logo of the upstream regions of 2,055 genes that are predicted to be either monocistronic or proximal genes in operons with an intergenic distance to the adjacent gene of at least 40 nt. (B) Sequence logo of the region upstream of 83 genes that are predicted to be distal genes in operons at a distance between 10 nt and 25 nt to the adjacent gene. (C) Sequence logo of the region upstream of 618 genes that are predicted to be distal genes in operons but less than 10 nt apart from the adjacent gene.

Experimental characterization of a limited number of genes revealed that the majority of transcripts were found to be leaderless and that leaderless as well as leadered transcripts lacking a SD sequence were efficiently translated in vivo. To address the question whether these results can be generalized, a bioinformatic analysis of the H. salinarum genome was performed (the H. volcanii genome is not yet annotated and thus could not be analyzed). The H. salinarum R1 genome guarantees a very high quality of translational start point annotation, because many proteomic data and gene homology data have been integrated (Pfeiffer et al., unpublished data; http://www.halolex.mpg.de). First, a genome-wide operon prediction was performed. Two adjacent genes were regarded as co-transcribed if they had the same orientation and their intergenic distance was smaller than 40 nt. This value was chosen because the distance between two monocistronic adjacent genes has to contain the termination signal of the first gene (i.e., at least a pentaU motif, Figure 3) as well as the promoter of the second gene (i.e., BRE + TATA box at −35 to −27, Figure 2). 2,055 genes were predicted to be monocistronic or proximal genes of operons and the regions upstream of their start codons were searched for conserved DNA or RNA elements. Figure 8A shows that no evidence for the existence of a SD consensus sequence was found in the region between −1 and −12. Instead, all three basal promoter elements were retrieved. Remarkably, the distance between the consensus sequences in Figure 8A and the translational start point is identical to the known distance between the promoter elements and the transcriptional start point, strongly indicating that the genes contributing to the consensus sequence have leaderless transcripts without any extra nucleotides upstream of the translational start codon. It should be pointed out that the information content is only 0.3 bits and thus this group contains also other transcripts, i.e., leadered transcripts and leaderless transcripts including one or a few upstream nucleotides. Nevertheless leaderless transcripts beginning at the translational start codon show the highest occurrence in this group of genes (Figure 8A).

Next, all predicted distal operon genes were analyzed. Initial attempts revealed that further subdivision was advisable. 109 genes had a distance of 26–40 nt to the adjacent gene. Unexpectedly, the generation of a sequence logo revealed that consensus sequences for the three basal promoter elements were present, but a termination signal from the preceding gene was missing (unpublished data). Therefore, this group of genes was seemingly transcribed into a bicistronic or polycistronic mRNA from the promoter of the proximal gene of the operon, and in addition, from a second promoter that is localized in the small intergenic region. This group is highly enriched in “hypothetical proteins” and “conserved hypothetical proteins” (67%, in comparison to the average value of 45%). 83 genes had a distance of 10 to 25 nt from the preceding genes. Generation of a sequence logo did not reveal the presence of any conserved element (Figure 8B).

A large group of genes (618) showed a distance of less than ten nucleotides to the preceding gene or had an overlap of up to ten nucleotides. Very faint overlapping signals with similarity to a SD sequence were detected, i.e., −14 GAGGUGA −8 and −12 AGGUGA −7 (Figure 8C). To reveal whether the signal from −14 to −7 in Figure 8C was due to SD sequences, 100 sequences were visually inspected and convincing SD sequences were indeed detected. The program “PatSearch” [36] was used for a systematic search for the occurrence of possible SD sequences in this group of 618 genes. As the minimal length of a functional SD sequence in archaea is not known, consecutive as well as non-consecutive matches of different length were retrieved, and the results are shown in Table 3. The spacing was set to a distance of 3–7 nt (the optimal distance determined for E. coli +/− 2 nt). Changing the spacing to a distance from 0–9 nt led only to a slight change of results (unpublished data). Consecutive matches of seven or eight nucleotides occurred nearly exclusively at the right distance and can be expected to be functional SD sequences. In contrast, four consecutive or five non-consecutive matches were found mostly in the wrong spacing and thus they do not seem to be sufficient to determine the translation initiation point. When the minmal requirement for a haloarchaeal SD sequence is set to ≥5 or ≥6 consecutive nucleotides that can hybridize with the 16S rRNA then 27% or 11%, respectively, of this group of genes is preceded by a SD sequence. Therefore the exact values vary with the definition of a SD sequence. Nevertheless, the analysis revealed that SD sequences do exist, but that only a minority of genes is preceded by a SD sequence even in this group of distal genes of operons. Due to the small intergene distances in this group the putative SD motifs overlap with the preceding open reading frame.

**Table 3:**
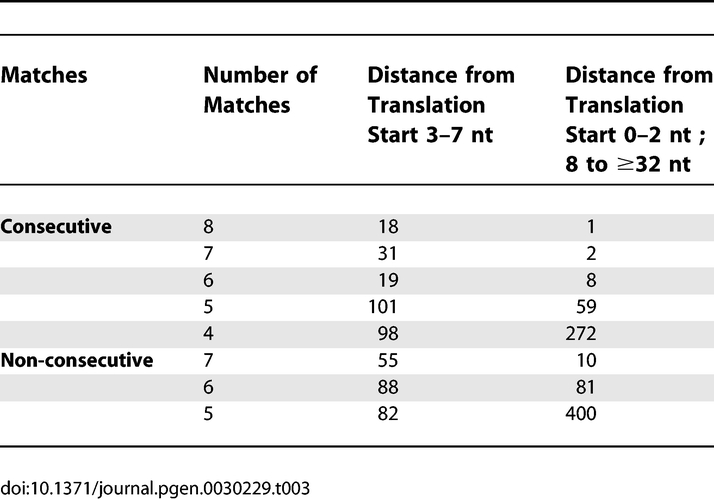
Occurrence of Strings Matching the SD-Sequence “GGAGGUGA” Upstream of 618 Distal Operon Genes of H. salinarum

The analysis was also performed with the group of 2,055 single genes and first operon genes and the results are shown in Table 4. In this case, 100 nt upstream of the start codon were used. Again, the data indicate that five non-consecutive matches do not function as a SD sequence because the vast majority is found at a wrong distance. The fraction of genes with five or six consecutive matches in the right distance is 3.6% and 1.6%, respectively, values that are considerably lower than in the group of distal operon genes. Taken together, the genome analysis revealed that a SD sequence plays a very minor role for translation initiation in haloarchaea, thus reinforcing the experimental results obtained with a limited set of genes (Figures 4–7).

**Table 4:**
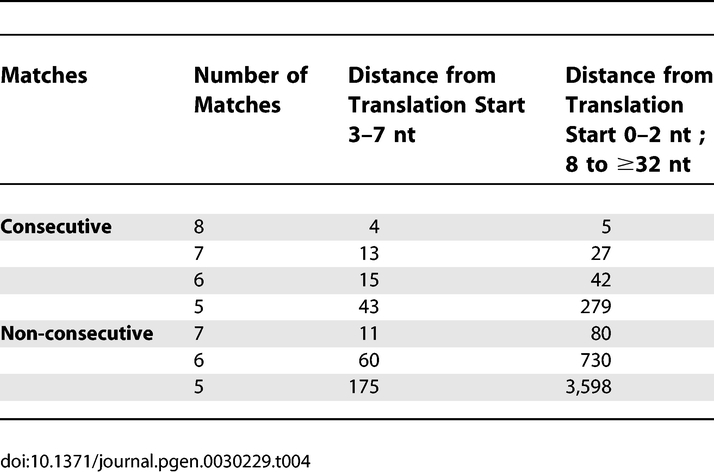
Occurrence of Strings Matching the SD-Sequence “GGAGGUGA” Upstream of the 2,055 Proximal Operon Genes or Monocistronic Genes of H. salinarum

## Discussion

### Determination of 5′- and 3′-Ends of Transcripts

We have determined the 5′- and 3′-ends of 40 haloarchaeal mRNAs using a recently developed method. In the original description of the method, Kuhn and Binder [28] came to the conclusion that only processed 5′-ends could be determined, but no primary 5′-ends. The experimental indication was that they found several different 5′-ends belonging to the single transcript analyzed. The theoretical explanation was that primary 5′-ends carry triphosphates which presumably cannot be ligated by RNA ligase. Therefore, for the determination of primary 5′-ends the triphosphates would need to be first converted to monophosphates using a pyrophosphatase [37]. However, this turned out to be unnecessary for haloarchaeal transcripts. In a proof of principle experiment the 5′-end of the *blp* transcript was determined, and the result was identical to an earlier study using primer extension (compare numbers 9 and 24 in Table 1). The results of the whole gene set yielded the following additional proofs that primary 5′-ends have been determined: (1) in all 40 cases a single 5′-end was discovered, and (2) the basal promoter elements (TATA box and BRE) had exactly the known distance to the experimentally analyzed 5′-ends, which can only occur when primary 5′-ends were determined.

The most conceivable explanation as to why the method worked so well with haloarchaeal transcripts is that a fraction of transcripts was dephosphorylated from the triphosphate to the monophosphate form. In E. coli it was shown that conversion of the 5′-triphosphate to a 5′-monophosphate is the first step in mRNA degradation. The fractions of 5-monophosphorylated cellular transcripts were quantified for two genes and found to be “18%–35%” and “up to half” [38]. If that would also be true for haloarchaea, it would explain the success of the method. However, it should be noted that for unknown reasons the method was not successful with some genes. Possibly, in these cases the treatment with a pyrophosphatase might have helped. However, either in the simple version applied here or in the version including a pyrophosphatase step [37], the method is very powerful and is expected to be extremely helpful for the determination of transcriptional and translational signals in a variety of archaeal, bacterial, and eukaryotic species.

### Promoter Structure of Haloarchaea

The regions upstream of experimentally determined start sites yielded consensus sequences for three basal promoter elements, i.e., the TATA box, the BRE, and a novel element at −10/−11. The same basal promoter elements were retrieved in a genome-wide analysis of the more than 2,000 genes, which are monocistronic or first genes in predicted operons.

The conserved region of the TATA box comprises only four (or five) nucleotides (Figures 2 and 8A). This is considerably shorter than the TATA box length of eight nucleotides determined in structures of TBP/DNA complexes from different species [39–41] and which is thought to be universally conserved in eukaryotes and archaea. However, two previous studies already reported that the consensus sequence for a haloarchaeal TATA box is shorter than eight nucleotides. A comparison of the regions upstream of experimentally determined 5′-ends available in 1999 revealed that the conserved region in haloarchaea was shorter than those in methanogenic archaea and in thermophilic archaea [2]. The in vivo selection of TATA boxes out of a random library of more than 10^6^ clones yielded a strong consensus sequence of six nucleotides [33]. The most probable explanation is based on the fact that haloarchaea have multiple TBPs, in contrast to all other species [42]. The TATA box consensus sequence of four nucleotides might comprise the common core that is detected by all haloarchaeal TBPs, while the remaining four nucleotides might be differentially recognized by individual TBPs. Thereby haloarchaeal TBPs could act as global regulators of transcription initiation, similar to bacterial sigma factors [42,43].

### Transcription Termination in Archaea

The results indicate that both a pentaU-motif at the very 3′-end of the transcripts and possibly a small stemloop near the 3′-end are involved in transcription termination. It should be pointed out that the putative stemloops are neither conserved in sequence nor in structure, therefore it is unclear how they could be involved in a general mechanism for transcription termination. Clearly, experimental data are needed to clarify whether the predicted stemloops are indeed involved in transcription termination.

A comparison with previous results is difficult since termination has not been thoroughly studied in archaea. The only report for a haloarchaeon revealed that the sequence “UUAUUC**UU**U” functioned as a terminator in vivo, while the mutated sequence “UUAUUC**GG**U” did not, confirming the importance of Us [43]. Some earlier studies with stable RNA genes from thermophilic archaea showed that termination occurred after extended oligo-U or oligo-pyrimidine stretches of more than ten nucleotides [7–9,44]. This might indicate that the oligoU-stretch required for termination is shorter in halophilic archaea, which have very GC-rich genomes, than in thermophilic crenarchaea. Recently one very thorough study of transcriptional termination in Methanothermobacter thermoautotrophicus was reported that used an in vitro termination system [10]. Natural and synthetic U-tracts were found to have the capacity and to be essential for termination while stemloops influenced termination in a way that could not be rationalized. Surprisingly, termination was also found to be influenced both by the topology of the substrate and by sequences up to 200 nt upstream of the termination site. Taken together, it appears that transcription termination in archaea does not have a uniform mechanism and that it is far from being understood. Determination of 40 haloarchaeal 3′-ends has identified a much higher number of in vivo functional terminators than in any other archaeal species and the results will guide further experiments.

### 3′-UTRs and Their Putative Biological Roles

Experimental determination of 40 3′-ends, a much higher number than in previous studies, revealed that all analyzed genes have a 3′-UTR with an average length of 57 nt. In eukaryotes, 3′-UTRs are longer and their length increased in evolution, e.g., 3′-UTRs of yeast and humans have an average length of 200 nt and 500 nt, respectively [13]. Eukaryotic 3′-UTRs are involved in a variety of different functions [12,13,16]. In archaea, the only function characterized previously was selenocysteine incorporation at stop codons within a few genes in some methanogenic archaea [34]. However, haloarchaea do not contain selenoproteins, and thus 3′-UTRs must have other functions. The reporter gene system showed that the addition of 3′-UTRs stabilized the *dhfr* transcript, and thus transcript stabilization is the second experimentally proven function of 3′-UTRs in archaea, which might be of very general relevance.

The 3′-UTRs are collinear with the genome sequence and posttranscriptional modification was not found to occur in any of the haloarchaeal transcripts analyzed. It has been shown that in exosome-containing archaea the 3′-ends are tailed post-transcriptionally with A-rich sequences important for RNA degradation [31,45]. However, Haloarchaea are devoid of an exosome and thus they have to use a different, as yet unidentified, RNA degradation pathway.

### Translation Initiation at Leaderless and Leadered mRNAs

The results of the experimental determination of 5′-ends of transcripts from two haloarchaea species as well as the bioinformatic genome analysis of H. salinarum revealed that the majority of transcripts are leaderless. Thus, this is regarded as the default pathway of translation initiation at least in haloarchaea. The characterization of a transcript as “leaderless” as well as the elucidation whether a leader contains a SD sequence (see below) critically depends on the correct annotation of translational start sites. For H. salinarum a high quality annotation of the genome was used, which integrates numerous proteomics results and cross-species comparisons (http://www.halolex.mpg.de; Pfeiffer et al., unpublished data). The translation initiation site of more than half of the leadered H. salinarum transcripts used in this study had been experimentally determined by peptide mass fingerprinting, and in the remaining cases it was verified that also possible start codons further downstream were not associated with SD sequences. For H. volcanii, codon preference plots were used to enhance the quality of correct start site selection [46].

Translation initiation on leaderless transcripts is brought about by a different mechanism than on leadered transcripts. In two archaea it has been shown that mutation of a SD sequence inhibited translation, while total deletion of the natural 5′-leader restored translation capacity [25,26]. In bacteria it has been shown that leaderless transcripts differ from leadered transcripts with regard to the requirement of initiator tRNA and translation initiation factors, the interaction with ribosomal subunits and the ribosome, and the resistance to the antibiotic kasugamycin. While translation on leadered transcripts initiates via an interaction with the small ribosomal subunit, bacterial and eukaryotic leaderless transcripts bind undissociated ribosomes and initiator tRNA to initiate translation [47–52]. Archaeal, bacterial, and eukaryotic in vitro translation systems are capable of translating leaderless transcripts, and therefore it has been proposed that this pathway is universally conserved and ancient [47,51].

As leadered transcripts are the minority at least in haloarchaea, their 5′-UTRs might have one or more biological functions in addition to translation initiation. The biological role of 5′-UTRs might be to repress translational efficiency compared to leaderless transcripts. This would allow fine-tuning of the protein amount produced per mRNA and it would enable the usage of differential translational control if positive regulators would enhance translational efficiency under certain conditions. 5′-UTRs could also be involved in (differential) regulation of transcript half-lives. Experiments to characterize the biological roles of 5′-UTRs in haloarchaea are underway.

It is currently believed that two major pathways of translation initiation exist, which are different in prokaryotes and eukaryotes. Prokaryotic transcripts require the interaction of a SD sequence with the 16S rRNA 3′-end, while in eukaryotes a scanning mechanism is used for translation initiation (in addition, exceptions exist like leaderless transcripts, internal ribosomal entry sites [53], and short upstream ORFs). The data presented here show that this does not hold true at least for haloarchaea, which use SD-mediated translational initiation only for a small subset of their genes. Evidences are (1) that most of the 19 leadered haloarcheal transcripts reported here do not contain a SD sequence, (2) that the seven experimentally characterized 5′-UTRs without a SD sequence mediate efficient translation in vivo (Figures 5–7), and (3) that the bioinformatic genome analysis indicates that SD sequences are not typical even for distal genes in operons.

The definition of a SD sequence is critical for the decision whether or not SD sequences are present in 5′-UTRs. In E. coli it is part or all of the sequence TAAGGAGGT that can hybridize to the 3′-end of the 16S rRNA. A bioinformatic analysis of all E. coli genes has revealed the consensus sequence AAGGA [54]. In most experimental studies, four to six consecutive nucleotides are used (e.g., [55] and references therein). The spacing is 5 nt +/− 2 nt between the 3′-edge of the aligned SD sequence and the start codon [54,55]. In contrast to the decades of research with bacteria only two experimental studies on archaeal SD sequences are available. Using a S. solfataricus in vitro translation system it was revealed that mutation of the native SD sequence **GAGGUGA** to the sequence **GA** C **GU** C **A** (nt with complementarity to the 16S rRNA are shown in bold, mutated nt are underlined) led to a complete loss of translation competence [25]. Note that the inactive mutant sequence retained five nt that could hybridize to the 16S rRNA. Using a H. volcanii in vivo system it was shown that mutation of the natural SD sequence **GGAGGU**C**A** to UU **AGGU**C**A** decreased translational efficiency by more than 90% although the mutant retained five SD nucleotides [26]. These results are in excellent agreement with the bioinformatic analysis of the H. salinarum genome. Four consecutive and five non-consecutive matches are found predominantly at wrong distances to the translational start point, and this is even true for five consecutive matches if the analyzed region is 100 nt (Tables 3 and 4). Taken together, bioinformatic as well as experimental results indicate that archaeal SD sequences might need more extensive base-pairing to the 16S rRNA for determining the translational start site than E. coli SD sequences. Even if a rather relaxed definition of “five consecutive matches” is applied, less than 10% of all H. salinarum genes use the SD pathway for translation initiation and more than 90% are either leaderless or use a novel mechanism devoid of a SD sequence.

It might turn out that a low occurrence of SD sequences and the usage of a non-SD pathway for translation initation is not confined to haloarchaea. A large-scale bioinformatic genome analysis predicted that 50% of all archaeal and all bacterial genes are not preceded by a SD sequence [56]. If that would be true, a high fraction of prokaryotic transcripts must either be leaderless or carry 5′-UTRs devoid of a SD sequence, in contrast to the current belief. It will be interesting to reveal (1) how widespread the occurrence of leaderless transcripts and of leadered SD-lacking transcripts is in different groups of prokaryotes, (2) whether translation initiation on leadered SD-lacking transcripts uses a scanning or a novel mechanism, and (3) how initiation on SD-lacking transcripts works mechanistically and to uncover the nature of the molecules involved.

## Materials and Methods

### Microorganisms, media, and growth conditions.

H. salinarum was provided from the German culture collection (DSMZ, Braunschweig, Germany, strain number DSM 670), *H. volcanii* WR 340 was obtained from Moshe Mevarech (Tel Aviv University, Tel Aviv, Israel), and E. coli XL1 Blue MRF' was purchased from Stratagene (Amsterdam, The Netherlands). H. salinarum was grown aerobically in complex medium containing 4.3 M NaCl, 80 mM MgSO_4_, 10 mM Na_3_-Citrate, 27 mM KCl, 1% (w/v) peptone (pH 7.2) at 42 °C [57]. H. volcanii was grown aerobically in complex medium containing 2.9 M NaCl, 150 mM MgSO_4_, 60 mM KCl, 4 mM CaCl_2_, 0.275% (w/v) yeast extract, 0.45% (wt/vol) tryptone, and 50 mM Tris-HCl (pH 7.2) at 42 °C [58]. E. coli XL1 Blue MRF' was grown in SOB Medium at 37 °C [59].

### Total RNA preparation and determination of 5′-ends and 3′-ends.

RNA was isolated from exponentially growing cultures as described by Chomczynski and Sacchi [60] or by using the RNeasy system (Qiagen) with DNase treatment according to the manufacturer's instructions. The quantity and quality of the extracted total RNA were determined by UV spectroscopy (at 260, 280, and 230 nm) and denaturing formaldehyde agarose gel electrophoresis [59,61]. RNA circularization, reverse transcription, and PCR amplification were carried out as described [28]. After denaturation at 65 °C for 10 min, total RNA was self-ligated by incubating 5–10 μg RNA with 40 U T4 RNA ligase (New England BioLabs), 10 U RNase Inhibitor (Promega), and 1 × T4 ligase buffer in a reaction volume of 25 μl at 37 °C for 1 h. After adjusting the total volume to 500 μl, proteins were removed by phenol chloroform extraction following standard protocols [59]. Self-ligated RNA was denatured and hybridized with 0.5 pmol of gene specific primer RT (see Tables S1 and S2) at 65 °C for 10 min. cDNA synthesis was carried out with M-MLV Reverse Transcriptase RNase H Minus point mutant (Promega) on 5–10 μg ligated RNA according to the manufacturer's instructions. The cDNA of the 5′-3′ ligated RNA was then amplified with gene-specific primer pairs PCR1/PCR2 followed by a second PCR with nested primer pairs NES1/NES2 (see Tables S1 and S2). The second, nested PCR enhances the specificity of amplification considerably and eliminates any possible false-positive fragments of the first PCR reaction. Nested PCR products of the 5′-3′ ligated RNA were subsequently analyzed by sequencing.

To analyze variable transcription stop sites, amplified nested PCR products were blunt-end ligated into the pBluescript vector pSKII (Stratagene) according to standard procedures [59]. Ten different cDNA clones were then analyzed by sequencing.

### Identification of conserved DNA and RNA sequence motifs.

The program “RNA structure logo” [29,30] allows us to identify and to visualize conserved sequence motifs that have a fixed distance to a point used for the alignment of a set of sequences, e.g., the transcriptional start site and the termination site. To create a logo, sequences with a defined length upstream and downstream of the alignment site were extracted from sequence databases, and the sequence logos were generated using the website: http://www.cbs.dtu.dk/~gorodkin/appl/slogo.html.

The program MEME [32] allows us to identify conserved sequence motifs in a set of sequences. The motifs can have variable localizations in different sequences, can occur in all or a subset of sequences, can occur 1- or several-fold in a sequence, and can be present in either strand. The website http://meme.sdsc.edu./meme/meme.html was used for the analysis.

### Prediction of secondary structures of complete RNAs, 5′-UTRs, and 3′-UTRs.

Three different programs were used for the prediction of the possible secondary structures of complete transcripts, 5′-UTRs and 3′-UTRs. The program “Mfold 3.2” [62,63] was used at the website http://www.bioinfo.rpi.edu/applications/mfold/rna/form1.cgi and the program “RNAshapes” [64,65] was used at the website http://bibiserv.techfak.uni-bielefeld.de/rnashapes. Dirk Metzler (Bioinformatics, University of Frankfurt) was so kind to fold the RNAs using a self-developed program that can take pseudoknots into account (unpublished data).

### Construction of plasmids containing 5′-UTR and 3′-UTR reporter gene fusions.

The shuttle vector pSD1/M2–18 [33] was used for the generation of a reporter system for the in vivo function of 5′-UTRs and 3′-UTRs. It contains replication origins and resistance genes for E. coli and H. volcanii as well as the *dhfr* gene under the control of a constitutive promoter of medium strength, which allows detection of both up- and downregulation of expression levels.

First, a control plasmid was constructed. The promoter region and the open reading frame of the *dhfr* were amplified separately by PCR reactions (the oligonucleotides for the construction of all plasmids are summarized in Table S3). The two PCR fragments were fused into one by a subsequent third PCR reaction, and the resultant fragment was cloned into the basal vector using ApaI and KpnI. The newly generated plasmid, pMB1, is very similar to the basic vector pSD1/M2–18, but the native 3′-UTR of the *dhfr* and nonnative nucleotides at the 5′-UTR (used for cloning in previous studies) were removed. Joining of promoter region and ORF by fusion PCR instead of ligation has the advantage that not a single nonnative nucleotide has to be introduced.

The plasmids pMB3 and pMB6 (Figure 5, numbers 2 and 4) were constructed in a similar way. The promoter fragments and the ORF were amplified as separate PCR fragments and joined by fusion PCR. The 5′-UTRs were part of the primers (see Table S3). The plasmid pMB4 (Figure 5, number 3) was also constructed by the same method. The 3′-UTR and a KpnI site were part of the downstream primer for amplification of the ORF fragment. For construction of plasmid pMB7 (Figure 5, number 5) three PCR fragments were generated containing the promoter region, the ORF, and the 3′-UTR, respectively. The three fragments were joined into one fragment by two consecutive fusion PCRs. In all five cases, the final fragments were cloned into the shuttle vector pSD1/M2–18 using single ApaI and KpnI sites. The plasmids pMB14–17 (Figure 7, numbers 10–13) containing artificial 5′-UTRs of random sequence upstream of the *dhfr* reporter gene were constructed as described above for pMB2 and pMB3. Primer sequences are included in Table S3. The newly generated regions of all plasmids were verified by sequencing. Then they were used to transform H. volcanii as described previously [58].

### Determination of *dhfr* transcript levels.

RNA was isolated from exponentially growing cultures (2 ×10^8^ cells/ml) as described by Chomczynski and Sacchi [60]. DNA was removed from the samples by incubating 6 μg RNA with RNase-free RQ1-DNase (Promega) according to the manufacturer's instructions. The RNA was purified using the RNeasy Mini Kit (clean up protocol; Qiagen). For reverse transcription, 1 μg RNA was denatured and was incubated with 0.6 μg of random hexamers (0.2 mM dATP and dTTP, 0.3 mM dCTP and dGTP) for 10 min at 65 °C in 38 μl 1x buffer (Promega). After cooling on ice for 2 min, cDNA synthesis was started with 2 μl M-MLV Reverse Transcriptase RNase H Minus (Promega) at 42 °C. After 1 h, additional reverse transcriptase (1 μl) was added. The reaction was stopped after 2 h by heat inactivation at 80 °C for 5 min.

Each real-time reaction contained 1 μl or 1.5 μl of template, 0.8 μM of forward primer and reverse primer and 12,5 μl DyNAmo SYBR Green qPCR Master Mix (Finnzymes OY) in a final volume of 25 μl. *dhfr* transcript levels were determined with the upstream primer Dhfr-RT_f, and the downstream primer Dhfr-RT_r. As an unregulated control, the *ribL*10 transcript levels were determined with the primer pair RibL-RT_f and RibL-RT_r.

The real-time PCR included 10 min initial denaturation at 94 °C, 60 cycles of 30 s 94 °C, 45 s 65 °C, 60 s 72 °C, and a final extension of 5 min at 72 °C. At last, samples were heated to 99 °C, cooled to 60 °C in 0.5 °C steps and the melting point of each sample was determined. The real-time PCRs were performed in the “Rotor Gene 3000” (Corbett Research). Data analysis was performed using the software “Rotor Gene 6.35” (Corbett Research). For each sample the intersection to a threshold line in the early exponential phase of the reaction was determined (Ct value). Data analysis was performed according to Livak and Schmittgen [66]. The Ct levels of the control transcript *ribL*10 were used to normalize the Ct levels of the *dhfr* transcripts. The *dhfr* level of the chromosomal gene copy was determined using a strain carrying a plasmid without a *dhfr* gene (pNP10) [67]. It was less than one-third compared with the strains carrying a plasmid with a *dhfr* gene. The value was subtracted to quantitate the transcript level of the plasmid-encoded *dhfr* gene. The *dhfr* transript level of the strain containing pMB1, which encodes a *dhfr* without 5′-UTR and 3′-UTR, was set to 1.

### Determination of DHFR activities and of translational efficiencies.

H. volcanii was grown aerobically in complex medium with 0.35 μg/ml Novobiocin. 10 ml of cultures in exponential growth phase (2 ×10^8^ cells/ml) were harvested by centrifugation (2,800*g*, 4 °C, 20 min). The cells were washed once in basal salt solution (2.9 M NaCl, 150 mM MgSO_4_, 60 mM KCl, and 4 mM CaCl_2_) and were then resuspended in 2 ml of basal salt solution. The cells were broken by sonication (2 × 30 s, output control: 5, duty cycle: 50%; Branson sonifier 250). The extract was then centrifuged at 18,000*g* and 4 °C for 30 min, and the pellet was discarded.

Dihydrofolate reductase activity was measured in 1-ml volume containing 100 μl of cytoplasmic extract, 700 μl buffer (3 M KCl, 25 mM potassium phosphate, 25 mM citrate [pH 6.0]) 0.05 mM dihydrofolic acid (Sigma), and 0.1 mM NADPH (AppliChem). The oxidation of NADPH was determined at 340 nm and 25 °C with a Specord S600 photometer (Analytik Jena). The enzymatic activity was calculated using an extinction coefficient of 6.22 mM^−1^ cm^−1^. Protein contents were determined using the BCA Assay Kit (Pierce) according to the manufacturer's instructions, with BSA as standard, and used to calculate specific enzyme activities (nkat/mg protein). The DHFR level encoded by the chromosomal *dhfr* copy was determined using a strain carrying a plasmid without a *dhfr* gene (pNP10) [67]. It was less than one-third compared with the plasmid-encoded DHFR values. Nevertheless, it was subtracted to quantitate the plasmid-encoded DHFR level (Figures 5 and 7).

The translational efficiencies were calculated by dividing the specific enzyme activities with the transcript levels. Three independent experiments were performed, and average values and standard deviations were calculated.

### Construction of his-tagged fusion genes and determination of translational efficiencies in *H. salinarum.*

The fusion genes encoding the respective protein with a C-terminal hexahistidine tag were generated by the production of two overlapping PCR fragments, both containing the histidine codons introduced by the PCR primers, and their consecutive fusion into one fragment using “ligation PCRs.” All primers are summarized in Table S3.

For the construction of the gene shown as number 1 in Figure 7, one PCR fragment contained 476 nt upstream sequences of the gene OE3100F, the 5′-UTR, and the open reading frame. The second PCR fragment contained 560 nt downstream sequences. Both fragments were fused by PCR.

For the construction of the gene shown as number 2 in Figure 7, the 5′-UTR was deleted. The deletion was performed by generating two overlapping PCR fragments, which contained the 476 nt upstream region and the open reading frame, respectively, but were devoid of the 5′-UTR. They were fused by a PCR reaction, and the resulting fragment was fused to the 560 nt downstream region by a further PCR reaction.

For the construction of the gene shown as number 3 in Figure 7, one PCR fragment was generated that contained 430 nt upstream of gene OE2082F and the open reading frame, and a second PCR fragment that contained 502 nt of the downstream region including the full-length 3′-UTR. Again, both contained the primer-encoded histidine codons and were fused by a subsequent PCR reaction.

For the construction of the gene shown as number 4 in Figure 7, the first 27 nt of the 3′-UTR were omitted from the downstream fragment. The shorter downstream fragment (475 nt) was fused to the identical fragment containing the upstream region and the open reading frame described above.

In all four cases, the resulting PCR fragments were cut with BamHI and KpnI (the sites were introduced with the PCR primers) and ligated with a BamHI*-*KpnI fragment generated from vector pSD1/R1–6 [33]. This fragment contained the E. coli vector pSKII and a gene conferring Novobiocin resistance to haloarchaea. The resulting four plasmids were used to transform H. salinarum as described [58]. Novobiocin (0.4 μg/ml) was used to select for clones that had stably integrated the plasmids into the chromosome by homologous recombination.

The transcript levels were quantitated by qRT-PCR as described above for the *dhfr* gene of H. volcanii. The qRT-PCR protocol differed in two details, i.e., annealing was performed at 65 °C and the elongation phase was 60 s. The primers are included in Table S4. The 3′-regions of the reverse primers are complementary to the histidine codons. Thereby it is guaranteed that only transcripts are quantitated that give rise to the his_6_-including fusion protein. As an unregulated control, the transcript level of the *hstA* gene was used.

To prepare cell extracts for immunoblotting, 15 ml of cultures in exponential growth phase (2 ×10^8^ cells/ml) were harvested by centrifugation (3,200*g*, 20 min, 21 °C).

The pellet was suspended in 1 ml a. bid. to lyse the cells. Proteins were then precipitated according to the method of Wessel and Flügge [68], and the precipitate was dissolved in 25 mM Tris-HCl (pH 8,6). Protein concentrations were determined with the BCA protein assay reagent (Pierce). Solutions of bovine serum albumin (BSA) were used as standard. Before electrophoretic separation, one-quarter volume of 4× SDS sample buffer was added. The samples were incubated for 5 min at 95 °C and 80 μg total protein/lane was separated on standard sodium dodecyl sulphate (SDS)-polyacrylamide gels. Subsequently, the proteins were transferred onto nitrocellulose membranes (Protran BA 83; Whatman, Schleicher and Schüll) by semi-dry blotting and probed with a specific Tetra-His antibody (Quiagen) according to the manufacturer's instructions. Horseradish peroxidase-conjugated goat anti-mouse antibody (Sigma) was used at concentrations recommended by the manufacturer. Immunoreactive bands were visualized by chemiluminescence (ECL substrate). For quantitation the films were scanned and the pictures were analyzed with the software “ImageJ” (http://rsb.info.nih.gov/ij/index.html). The background was determined locally for each band and subtracted from the signal.

### Genome-wide in silico analyses.

The identifiers, positions of the start and stop codons, and the direction were retrieved from the genome database (http://www.halolex.mpg.de). Microsoft Excel was used to identify groups of genes that have the same direction of transcription and have intergene distances of at least 40 nt, 26 nt–40 nt, 10 nt–15 nt, and below 10 nt. For the first group of genes, the upstream regions from −1 to −100 were retrieved from the genome database, for the other groups the upstream regions from −1 to −40. For data analysis the programs “RNA structure logo” [29,30] and MEME [32] were used as described above. The program “PatSearch” [36] was used for the identification of putative SD sequences in the four groups of genes. The search pattern was varied, e.g., both consecutive as well as nonconsecutive matches to the SD sequence “GGAGGUGA” were retrieved and the length of the search string was changed. The searches were performed (1) in the 40 nt upstream of the 618 genes that are distal genes of operons and have a distance of less than 10 nt to the preceding gene and (2) in the 100 nt upstream of the 2,055 single genes or proximal genes of operons. The results tables were sorted to derive the number of genes with matches in a distance of 3–7 nt from the translation start and the number of genes with other distances. From the results tables that contained the number of genes with “at least x matches” the number of genes were calculated that contain “exactly x matches,” with “x” being an integer between 4 and 8 as shown in Tables 3 and 4.

## Supporting Information

Table S1 Primer Used for 5′- and 3′-End Determination of Transcripts of H. salinarum
(50 KB DOC)

Table S2 Primer Used for 5′- and 3′-End Determination of Transcripts of H. volcanii
(46 KB DOC)

Table S3 Primer Used for Reporter Plasmid Construction and Real-Time PCR (77 KB DOC)

Table S4 Primer Used for Construction of his-Tagged Fusion Genes and Real-Time PCR (37 KB DOC)

## Abbreviations

BRE: B recognition element
qRT-PCR: quantitative reverse transcriptase PCR
RT: real-time
SD: Shine-Dalgarno
