# Supplementary material for: Experimental Characterization of Cis-Acting Elements Important for Translation and Transcription in Halophilic Archaea
Source: PLoS Genet. 2007 Dec 21;3(12):e229. doi: 10.1371/journal.pgen.0030229 (PMC2151090; doi:10.1371/journal.pgen.0030229)
Supplement: Supplementary file 1 [file pgen.0030229.st001.doc]

| Table Supp-1: Primer used for 5´- and 3´-end determination of transcripts of *H.salinarum* | | | |  |  |
| --- | --- | --- | --- | --- | --- |
|  |  |  |  |  |  |
| **Identifier** | **RT Primer** | **PCR1 Primer** | **PCR2 Primer** | **NES1 Primer** | **NES2 Primer** |
| **OE3538R** | CCTGGCGTGCTGGTTGGTTGC | AAGGAAGTCACCCGTGTGCCGTTC | ACGGGATCGGCTTCCGCTGG | CGTGTGCCGTTCGCCGAACCC | TGGACACGGCAGGCTGTCGCC |
| **OE4187R** | CTTCCTCGTCGAGGGCCTCGTAG | GACGACCTCCTCGCCGACGCC | GCCTCGTAGAAGTCGGAGGCAACG | ACGCCGCGGAACGCGCCGAG | CGGAGGCAACGTTCTTGTCGTCG |
| **OE2082F** | TGTCGAAGTATCGGAGGCCGTCG | ACCCGTTTTGCGCGTACGGGG | CGGCGGGATCGGTGACTGGTCG | CGTTCTCGTGTGCGCTGCCGC | CTCGTCTTTGTCCGCGCGGTGG |
| **OE2024F** | TGTCAGCGAGCTCCGGCTTCG | ACACCGGCCACACCAGCCTCG | GTCTCGGACTGCCCGCATGAGG | ACACCGACGACGCCCAGGCTGTG | GCTGCGCGAGCAGCTGGAACAGC |
| **OE1982R** | ACAACAGCATGCCACTGACCGCG | ATCTACACGGGGACGACCGCCAC | CGAGTCCGACCTCGGTGACGACG | ACCCGCGGGATCGCGATGCTC | TCACGAGCGGGATCGGCCACG |
| **OE3090R** | TCGGACACCGTGAGTTGCGCG | GAGATGCAGCTCACGCCGCCC | CACGTCGTCGTCGATCATCGCCG | ACGCCACCCGACGCCGTTCG | CGAACACGCCGCCGAGACTGTC |
| **OE1147R** | ATGCCCCGCGTCGACGAACTC | GTCGAGCGCAACCGCACCGTG | GCGGGGAACCGCACGCATCG | GAGGACCGCGAGCGCGTGCG | CGCGTCGAGGAGGCTGTCGACC |
| **OE1405R** | CCGTGGTTGACCTCCTCGCCG | GCTTCGACGCGGTCGTCGACG | TCGAGCTCGCGCTTGATTGCGAG | CTCGAAGTTCGTCGCCGACGCC | GGCTTCCTCGCCGAGGACGGTC |
| **OE3100F** | GCTCGTTTGCCGCCACTGGC | GACCTGCCGGACACGGTCATCG | GCGCTTCGACATCCGTGACGGTG | TGCTGGCCCCGATCGCAAAAGC | ACGTGGTTCCGAGGGTCATCGAGG |
| **OE3049R** | CGGATCGACGGTTTCGGTGTCC | GCCCCGACGAGCTCAGCATCGAG | GCCGACAGCGTGATCGTGTCGTTG | CGAGCTGGAATGGGACGACGCC | TAGGTTGTCCGCGACGGTGCGC |
| **OE3470F** | CCACGTCCGGTATTTCATGCGGC | CTCGCCGAGCCGTGGGACTGG | GCGTCCCATCCGCTCTTTGCCG | CGGTACTCCGACCAGGACGCC | CCGCCGTCGTGTCGCTTGACG |
| **OE1765R** | CGTTGGCATCAAGCAGCCGGTC | GTTCGCCATCCGCGACGACCG | GCAGCCGGTCTCGCATCTCCTCG | AGGACTTCCAGTCGGCACGCGAG | GTGAGGCGCTGTCGTCGTAGGGG |
| **OE4136R** | TTTTGCATCCGGCCGGGGATG | CATCGTGCTCGTGGAGCCCTGG | TCCGCACACCGCACCTGCACG | AGGACGACAAGGCCGACGTGACG | CGAGCATGTCCGTGACCTCCGCG |
| **OE3934R** | CACTTCCTCGTAGCTGGCGGTGC | GGGTCGGCCACGTCCTCGAACAG | TACACGAGCTTGCCGGCGTCGC | CGACAACCGCCTCATCCGACCG | CGCCGTCGATGAAGCTCAGGTCG |
| **OE2186R** | CCTTCTGGAACTCGCCGATCGCC | AAATCCGAGTCCGCGCCCGACG | CGGGATCTTGTTCGCCCCGAACAG | CCGACACGACCAGCGACACGACC | TGGCGATGAGCAGGACGACCGC |
| **OE4583F** | TCTCCGGCACTGTGGACGCACG | CCGGCACTCGAACAACGAGTCGC | CCTGTGCGATGTCCTCCGCGGG | GTCGCTGGGTGACGCCGAGATCG | CGGCTTGGATGAGCCCTTCGCG |
| **OE4674F** | GGTCGCTGTCCCCCCGATAGAAC | GGGACGCTTGATCCGTCGCCGATC | CACAGACCGCCGTGTGGGTCACG | TCGACCTCGACGGTGTCCCGGAG | CGTCCTCGGGCGACTCGATGTCG |
| **OE2935R** | CCTTCTTGACGACGCCGAGCGC | CACGGAGGTCGTCGTCGAGAAGGG | CGAACGCGATCCCCGAGATCGGG | ATACCTCTCGGAGGCGGAGGCCG | TCGATCGCGCGCTGGGTCTGTG |
| **OE3817R** | CGGGTCGCCCAGAAGTCAGACTG | CAACAACACGACCACGGCCGCG | GCCCGTCTTCGCGTAGTTCGCCC | GCCGCAGCCTCCTCGACGAGACC | TCGAGTCGCGTTTCGAGGTCCGC |
| **OE4412R** | TAGTCGGCGTACCGCGCCTCC | GCGCGTGGTCTGTCTGACCACCG | ACACCACGTCCGCGAACGGTGC | GGCCACCTGCTGAAAGACCCGGC | CACCACACTCCACGCACGCCAGC |
| **OE1972F** | CGGGCGTGTAGAGCCGATCGAC | ACCATCCCGTACATCGTGGCCGC | GGTCGCAAACCGGGCTTTGCG | GGTCAAGGCGTACGCCACCATGGG | CTCGCCGTACGCGTCCTGCACC |
| **OE3717F** | CACGATGCGGGTGACGTCCGC | GTGCCGACCAGTACCGGTCGCTG | GTGCGAACGCCGTCACGTCGTG | GCCAACGACGTGACCGCGTTCG | GTGTGCCGATGAACCCCGTCCCG |
| **OE2906R** | GTCTCACGGTTCTCCGCCAGCG | ACTACGACTACGGCCCCGACCGC | AGGCCGTCCACGTAGCTCTGGTGG | GCGGCAGCTTCGTCGACGCCTTC | GGTGCCACGTGACCACCTGCTCAC |
