# Supplementary material for: Experimental Characterization of Cis-Acting Elements Important for Translation and Transcription in Halophilic Archaea
Source: PLoS Genet. 2007 Dec 21;3(12):e229. doi: 10.1371/journal.pgen.0030229 (PMC2151090; doi:10.1371/journal.pgen.0030229)
Supplement: Supplementary file 2 [file pgen.0030229.st002.doc]

| Table Supp-2: Primer used for 5´- and 3´-end determination of transcripts of *H.volcanii* | | | |  |  |
| --- | --- | --- | --- | --- | --- |
|  |  |  |  |  |  |
| **Identifier** | **RT Primer** | **PCR1 Primer** | **PCR2 Primer** | **NES1 Primer** | **NES2 Primer** |
| **FS451E8** | GGTCAGCAGGTTCATGTCCGTCTC | GCGAGTTCCTGCGGGAGTACGTC | GATGGTCTCGGGCTCGACGAACG | ACGTCCGCGATCACGGCATTCTG | GAGATGTCGTCGCGCTCTCGCAG |
| **FS437B8** | ATAACCGAGAGCGCCTCCTCACC | GACGCCGTGAACACGAGGGATAC | TGCGGTTTCGACCTCGCAGTAGC | GCCGGACGACAGCATTCTCGACC | GCTACTTCCCGCTCGTCGTCCAC |
| **FS448H7** | AGGTCGTCGTCGGAGAATCG | CCGGCGTCTCCCACGGGTTC | ACGAAGTGCGGCGCGTAGAC | GTTCGCCCACCTCGGCTACG | CGTCGATGCCGCGGGCTTTC |
| **FS452E1** | GTCTCGGTCGTCGATTCGGTCTC | GCGCCAAGCAGTTCTATCCCGAG | GAACCGCCCTCTCCGGTACAGCC | CCGAAATCCCGGAAGACGAACAG | CCGCGCCGCCGTACTTCACGTAG |
| **FS436G6** | GTGAGCGTGGACGGTGCTCTTC | TGAGCGGTCCCGCCTACGTCATC | TCGCCTCGACCAGTTCGCCCATC | TTCGAACAGGGCGTCGCGGAAC | TGTGGTCGACGAGCGCCAAGGAG |
| **FS433H6** | AGTCGTCTTCGTCGTCGGGTTC | ACGCCGACTTCGACTCGTGGGTG | GTGCCGCTCGCACCGGTCAC | CGTCCTCGCGCTCTGTTCGG | CCGGTCACGAGAGCGACGGTTCC |
| **FS459C6** | GGCGTCGAGGGAACTGAACAC | CGGCCTCAGCGTCGTCATGAGC | GCGCACGTCTCGGTCGTGAACCC | CGGCTGGAGCGTCTGGTTCGTC | TCGTGAACCCGCGTCGTCCGAG |
| **FS431G8** | ACGAGCGGTCGCTCGATG | CGGACGACGGTCACGACCACAG | GTCCTCGGCGTCCGCCCAGAAG | ACCACGACCACACGGACGAACAC | CGTCAAGCGTTCGGTCGTCAGTG |
| **FS458C1** | GCCGTGCTGCTCGTCGTAG | CGCTCCTCGCCGACGAGGAACTG | ATTCGGTCTCGGCCTCGCCCTCG | ACCGCTACCGCGAACTCC | GACTGTCGGGCTTCGGGTCC |
| **FS431F1** | TACTGCCGTCTTCGGTCGTCTGC | GGCGACACGCAGGACTTCTC | TGCCCGGAACTATCGAAGCAGCC | ATGCCGACGGGCGACGACTACG | ATCGAAGCAGCCGGCCAGTCCAG |
| **FS451C1** | ATGCTCGGCCACGGGAGTTC | CCGCCGAGTGGGAACTCGAC | CGGCCGATGACGCGGTTCTC | AACTCGACGCCGAGACCGACCAC | GCGGCGACAGAGACGAGTTCCATC |
| **FS436F5** | GAGCGTCCACGGGAAGTTGC | CACTCCTACGAGCTGGGCTGGTG | TTCGCCGTCACGCCCATCGTCTC | CCTCGGCGTGACGCTGGACATGG | CTCCGCCGACAGCTCTCGCATCGTG |
| **FS434E1** | CCGTCCCGATGAACAGGAGGAG | ACGAGGTGTCGGTGCGGGAGTTC | GGACGCGGTAGAACAGAATCCAGC | GTACGCCTTCGGCGCGGGACTCG | CCGACGACGGCGAGAACGACCAC |
| **FS441B9** | CGAAGAACCGTCGCGAGAGG | CGGGCTCCAGCTCGCTGAACTC | CACGTTCGCACGCCGGAGCTTC | CGAGAAGCAACTGCTCGCCATCG | CGGTGCTTCGGCCGCAGAGTTC |
| **FS456E3** | GGACCGCCAACATCTTCCATGTG | CTCGAAGAGGGTCAGGAAGTG | AACACGTCGTCGTCCGCGTCGTCAG | GGGTCAGGAAGTGGAGTTCGACATC | CGTCCGCGTCGTCAGTAGAGATG |
| **FS456E4** | AGCCGCCAGTGTCGTTGAAGAAG | GACGACGACGTGTTCTTCC | CGCCATTGCCTTTGAAACCAACG | ACCTCGAAGAGGGTCAGGAAATC | CGACCTCACGCTGATAAGTCTTC |
| **FS456E5** | TCGCCTCGTCGTCGGTCGTC | GGTTAGTCGTCGAGCCGGTCCAG | GCCGAACCGCGAGGAGGTACGTC | GGAACGGAAGATGCTCAAAGG | GTACGTCCCGACGACGCCGATTCC |
