# Supplementary material for: Experimental Characterization of Cis-Acting Elements Important for Translation and Transcription in Halophilic Archaea
Source: PLoS Genet. 2007 Dec 21;3(12):e229. doi: 10.1371/journal.pgen.0030229 (PMC2151090; doi:10.1371/journal.pgen.0030229)
Supplement: Supplementary file 4 [file pgen.0030229.st004.doc]

Table Supp-4: Primer used for construction of his-tagged fusion genes and Realtime PCR

| **Name** | **Sequence (5´ 3´)** | **Application** |
| --- | --- | --- |
| **3100-His-ORF-Linker** | TCAGTGATGGTGATGGTGGTGGTGTTCGAGGAACGTCGTGAGG | amplification of the orf of OE3100F ,addition of the his-tag and fusion to the 3´region |
| **3100-His-3´UTR-Linker** | CACCACCATCACCATCACTGATCCGCCACAGCCGGCGTGTG | amplification of the 3´region of OE3100F, addition of the his-tag and fusion to the orf |
| **3100-Promotor-Linker** | CGACCGTGAACATGTGTGGATGATGGTGGCGTGGGTG | amplification of the orf of OE3100F and fusion to the 5´Region |
| **3100-ORF-Linker** | CCATCATCCACACATGTTCACGGTCGAACAACAGGCC | amplification of the 5´region of OE3100F and fusion to the orf |
| **3100-BamHI** | CGGGATCCCGGTGCCACGGTGAGCTCGG | amplification of the 5´region of OE3100F and addition of a BamHI restriction site |
| **3100-KpnI** | GGGGTACCCCCAGGCGGTCGTCGACAC | amplification of the 3´region of OE3100F and addition of a KpnI restriction site |
| **2082-His-ORF-Linker** | TCAGTGATGGTGATGGTGGTGGTCGGCGTCGACGCGCTCCC | amplification of the orf of OE2082F ,addition of the his-tag and fusion to the 3´region |
| **2082-His-3´UTR-Linker** | CACCACCATCACCATCACTGAATCGGCGGTCCACGGGACGCG | amplification of the 3´region of 2082F with complete 3´UTR, addition of the his-tag and fusion to the orf |
| **2082-His-Term.Linker** | CACCACCATCACCATCACTGAAGGCGGCGTCAGGCGGCTGC | amplification of the 3´region of 2082F with truncated 3´UTR, addition of the his-tag and fusion to the orf |
| **2082-BamHI** | CGGGATCCCGCAAGCGGCAGCAACGAG | amplification of the 5´region and orf of OE2082F and addition of a BamHI restriction site |
| **2082-KpnI** | GGGGTACCCCGGGCGCTCCATCGAGGG | amplification of the 3´region of OE2083F and addition of a KpnI restriction site |
| **3100-RT-f** | GTCGAAGACATCGAACGCGTGCCCGAG | amplification of OE3100F his (Realtime PCR) |
| **3100-RT-r** | GTGGCGGATCAGTGATGGTGATGGTGGTG | amplification of OE3100F his (Realtime PCR) |
| **2082-RT-f** | ACCTACGAGCACGGCCGCTACCTGGACG | amplification of OE2082F his (Realtime PCR) |
| **2082-RT-r+3´** | CCGCCGATTCAGTGATGGTGATGGTGGTG | amplification of OE2082F his (Realtime PCR) |
| **2082-RT-r-3´** | GCCGCCTTCAGTGATGGTGATGGTGGTG | amplification of OE2082F his with truncated 3´UTR (Realtime PCR) |
| **1220-RT-f** | CATCCAGCGTCATGGGGCGATCCTCG | amplification of OE1220F (Realtime PCR) |
| **1220-RT-r** | TCCTCGGACACCCGGAACCGGTCGTC | amplification of OE1220F (Realtime PCR) |
